# In vitro interaction profiles and midgut glycoconjugates of *Trichophoromyia* spp./*Leishmania* (*Viannia*) *lainsoni*

**DOI:** 10.1186/s13071-025-06860-6

**Published:** 2025-06-09

**Authors:** Thais Gouvea de Morais, Yetsenia del Valle Sánchez Uzcátegui, Chubert Bernardo Castro de Sena, Rodrigo Ribeiro Furtado, Fernando Tobias Silveira, Edilene Oliveira da Silva, Thiago Vasconcelos dos Santos

**Affiliations:** 1 Programa de Pós Graduação em Biologia de Agentes Infecciosos e Parasitários, Instituto de Ciências Biológicas, Universidade Federal do Pará, Belém, Pará Brazil; 2 Seção de Parasitologia, Instituto Evandro Chagas, Ananindeua, Pará Brazil; 3 Departamento de Biología, Faculdad de Ciencias, Universidad de los Andes, Mérida, Venezuela; 4 Laboratório de Biologia Estrutural, Universidade Federal do Pará, Belém, Pará Brazil

**Keywords:** Sand fly, Attachment, Glycoconjugates, Vector, *Leishmania*

## Abstract

**Background:**

Knowledge of sand fly–*Leishmania* attachment determinants is pivotal for providing evidence on vector status. Considering the Amazonian transmission context of *Trichophoromyia* spp.–*L*. (*Viannia*) *lainsoni*, the present study aimed to assess in vitro interactions and detect gut glycoconjugates associated with this vector–parasite association.

**Methods:**

Field-caught *Trichophoromyia brachipyga* and *Trichophoromyia ubiquitalis* were tested. *Lutzomyia longipalpis* reared in the laboratory was used as a control. The intestines were obtained by dissection, and the species were confirmed by morphology. Interactions for each sand fly–*Leishmania* association were individually performed via an in vitro incubation system. *N*-acetyl-d-glucosamine (GlcNAc), galactose-(β 1,3)-GalNAc (Gal/GalNAc), and *N*-acetyl-d-galactosamine (GalNAc) glycoconjugates of *Trichophoromyia* spp. were analyzed by Western blotting using corresponding peroxidase-conjugated lectins.

**Results:**

No difference was found between *Th. ubiquitalis* and *Lu. longipalpis* attachment with *L.* (*V*.) *lainsoni*, and *Lu. longipalpis* with *Leishmania* (*Leishmania*) *infantum* (control). However, *Th. brachipyga*-*L*. (*V*.) *lainsoni* attachment was weaker than that of the control. *Trichophoromyia* spp. were negative for residues of GlcNAc terminally exposed. *Trichophoromyia ubiquitalis* was positive for GalNAc and Gal/GalNAc, whereas *Th. brachipyga* presented only residues of GalNac terminally exposed.

**Conclusions:**

The present study suggests that *Trichophoromyia* spp. sand flies, particularly *Th. brachipyga*, are susceptible to *L.* (*V.*) *lainsoni*, based on the observed vector–parasite attachment profiles and detection of GalNAc in their midguts. This supports early field data suggesting the vector status of these sand fly species.

**Graphical Abstract:**

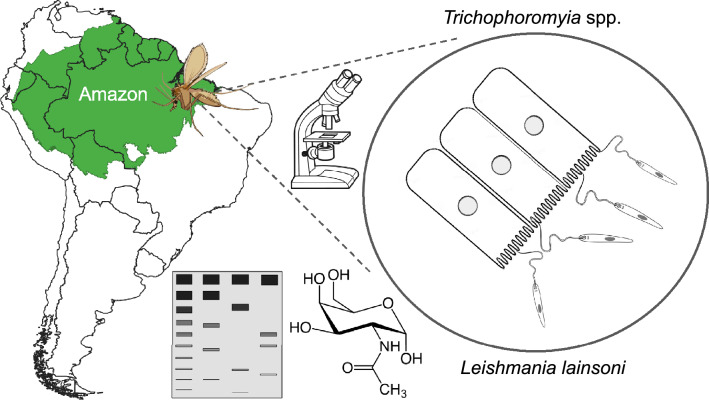

## Background

Cutaneous leishmaniasis (CL) is a neglected tropical disease with a wide geographic distribution and can cause severe clinical manifestations [1]. In the Amazon region, a myriad of parasite–vector–host arrangements reflect a complex network of interactions [2]. Despite their medical and epidemiological relevance, some transmission cycles remain understudied, which limits the effectiveness of specific surveillance strategies.

*Leishmania* (*Viannia*) *lainsoni* was first described in the 1980s in the state of Pará, Brazil, as a causative agent of cutaneous leishmaniasis in humans [3]. Since then, it has been identified as a causative agent of the disease in other Brazilian states and neighboring countries [4–10]. *Trichophoromyia* species have been associated with the transmission of *L.* (*V*.) *lainsoni* [11]. Whereas *Th. ubiquitalis* has long been regarded as a vector of this parasite [12, 13], *Th. brachipyga* has recently been included in the suspicion list on the basis of a natural infection diagnosis [11, 14].

Attachment to the sand fly midgut is a crucial step for *Leishmania* survival in the vector and is included in the determinant set of vector competence [15, 16]. Carbohydrate epitopes, primarily *N*-acetyl-d-galactosamine, present on the midgut microvillar surface, bind to *Leishmania.* [17, 18]. Furthermore, under laboratory conditions, the attachment capacity of a given sand fly to one or more *Leishmania* species can be classified as specific or permissive, respectively [19]. In vitro attachment experiments have been performed to allow rapid analyses of the interaction between insect midguts and promastigote forms of *Leishmania* [18, 20–28], providing alternative evidence of that in vivo, where unproductive offspring and unsuccessful artificial blood-feeding of wild-caught species may limit sample size [29, 30].

Given the epidemiological relevance of CL agents in the Amazon region and the limited knowledge of sand fly–*Leishmania* interactions, studies addressing these gaps are essential. This work aimed to evaluate the in vitro interaction between *Trichophoromyia* spp. and the associated parasite *L.* (*V.*) *lainsoni,* as well as to detect glycoconjugates in the sand fly midgut.

## Methods

### Sand flies and* Leishmania* spp.

The field-caught sand flies used in this study were obtained from an urban park located in the Belém Metropolitan Region, Pará State, Brazil (1° 25′ 48.2′′ S, 48° 27′ 24.9′′ W), where the ecology of these insects has been previously studied; in that environment, *Th. ubiquitalis* and *Th. brachipyga* are abundant [14]. Entomological capture was performed using Centers for Disease Control and Prevention (CDC) light traps (John W. Hock Company, Gainesville, USA) installed between 6:00 pm and 6:00 am, close to the ground (~1.5 m). The sampling effort was not established, as it was based on the availability of specimens, the success of the experiments, and, consequently, the statistical support for the findings.

An old, closed colony of *Lutzomyia longipalpis* from the 1990s (Abaetetuba, Brazil, F245) was used as a control, as this species is recognized as a permissive vector [17, 19]. The insects were reared and maintained under a 14:10 h light/dark regime at a temperature of 25 °C, a relative humidity of 80%, and a diet of 10% sucrose. Previous evidence has demonstrated that this species is experimentally susceptible to *L.* (*V*.) *lainsoni* [3].

Promastigotes of *L.* (*V.*) *lainsoni* (MHOM/BR/1981/M6426) and *L*. (*L*.) *infantum* (MCER/BR/1981/M6445) were obtained from the *Leishmania* cryobank of the Ralph Lainson leishmaniases lab (Instituto Evandro Chagas, Ananindeua, Brazil) and maintained in Roswell Park Memorial Institute (RPMI) culture medium supplemented with inactivated 10% fetal bovine serum (FBS) at 25 °C to estimate the growth curve.

### Dissection of midguts

Field-caught samples were immediately transported to the laboratory in a nylon cage as described elsewhere [29]. Insects were placed in a container, immobilized by cooling (4 °C), and then successively washed with phosphate-buffered saline (PBS) [31]. The sand flies were morphologically screened to triage the target species. Females were dissected under sterile conditions in Grace’s insect medium (Merck, Darmstadt, DE) within concave wells of glass slides with the aid of entomological forceps following decapitation and removal of the last abdominal segments to obtain the spermatheca and digestive system [28]. The spermatheca was placed on a slide and covered with a coverslip to confirm the species under an optical microscope, following the taxonomic identification keys of Galati [32]. After taxonomic determination, the midgut and Malpighian tubules were removed, and the midgut was retained. These procedures were continuously performed to ensure sufficient material for the experiments described in the following sections.

### In vitro interactions

The following sand fly–*Leishmania* associations were considered: *Th. ubiquitalis*–*L*. (*V*.) *lainsoni* and *Th. brachipyga*–*L*. (*V*.) *lainsoni* as tests and *Lu. longipalpis*–*L*. (*L*.) *infantum* and *Lu. longipalpis*–*L*. (*V*.) *lainsoni* as controls.

Late logarithmic phase promastigotes were centrifuged at 1800 × *g* for 10 min and resuspended in 1 mL of Grace’s insect medium (GIM) [28]. A dilution for counting was subsequently performed in the Neubauer chamber to estimate and subsequently adjust the inoculum to 2 × 10^7^ cells/mL [25].

The midgut was dissected, and the remaining structures were removed as previously described. The concave wells of glass slides containing 5 μL of GIM were longitudinally opened and incubated for 45 min with 50 μL of promastigotes (2 × 10^7^ cells/mL) in a dark and humid chamber. After incubation, the midgut was washed three times in PBS using two pairs of needles and syringes (one pair to remove PBS from the slide and one pair to add sterile PBS again) to remove promastigotes that did not adhere to the intestinal epithelium [25, 26]. After washing, the well of the slide containing the midgut was covered with a coverslip and observed under 400× magnification with an AxioScope optical microscope (Zeiss, Oberkochen, DE). The observed adhesion profile was classified as weak (fewer than 100 promastigotes), moderate (100–1000 promastigotes), or heavy (more than 1000 promastigotes), adapting the criteria of Myskova et al. [17] for in vitro interaction assays. Each combination of sand fly–*Leishmania* was evaluated in 40 dissected midguts.

### Detection of glycoconjugates

Previously standardized samples of seven pooled midguts were stored in microtubes containing 25 μL of cell lysis buffer (20 mM Tris–HCl pH 7.5, 150 mM NaCl, 1 mM Na_2_-Ethylenediaminetetraacetic acid (EDTA), 1 mM ethylene glycol tetraacetic acid (EGTA), 1% Triton) with protease inhibitor cocktail (Complete, Mini EDTA-free) (Roche, Basileia, CH) at 4 °C. The midguts were then macerated, heated to 100 °C for 5 min, and then frozen in liquid nitrogen until the experiments. Subsequently, samples were thawed and 15 μL mixed with 5 μL 4 × SDS sample buffer (final concentration: 250 mM Tris–HCl pH 6.8, 40% glycerol; 8% SDS; 20% 2-mercaptoethanol; 0.2% bromophenol blue) and heated at 100 °C for 3 min using a Thermo-Shaker (KASVI, Pinhais, BR). After that, the samples and molecular weight marker were applied to a 10% polyacrylamide gel for the separation of glycoconjugates by sodium dodecyl sulfate–polyacrylamide gel electrophoresis (SDS-PAGE; 120 V for 1 h) using a Mini-Protean Tetra chamber (Bio-Rad, Hercules, USA) [17, 18].

Then, the transfer to a nitrocellulose membrane was performed using the ENDURO™ Semi-Dry Blotter (Labnet, Edison, USA) at 15 V for 30 min. To block nonspecific sites, the membrane was incubated overnight with skim milk/tris-buffered saline-Tween (TBST) (0.05 g/mL) at pH 7.6. After blocking, the nitrocellulose membrane was incubated for 1 h with the lectins from wheat germ agglutinin (WGA) (Sigma-Aldrich, St. Louis, USA), peanut agglutinin (PNA) (Sigma-Aldrich, St. Louis, USA), and *Helix pomatia* agglutinin (HPA) (Sigma-Aldrich, St. Louis, USA), conjugated with peroxidase in TBST (1:100) to determine GlcNAc, Gal/GalNAc, and GalNAc, respectively. The membrane was washed three times with TBST followed by incubation with 3,3′-diaminobenzidine tetrahydrochloride (Sigma-Aldrich, St. Louis, USA) for a few seconds until the bands could be visualized. Carbohydrate labeling was analyzed using ImageJ 1.5a software (National Institutes of Health, Bethesda, USA) [17, 18].

### Data analysis

To analyze statistically significant differences between in vitro interactions, the G test was performed for two independent samples with categorical variables. The confidence interval was set at 95%. Tests were performed via Bioestat 5.0 software (Instituto Mamirauá, Tefé, Brazil) [33], and graphical elements were created via Prism 10.3.1 (GraphPad Software, Boston, USA).

## Results and discussion

### Sand fly–*Leishmania *in vitro interactions

Each of the four sand fly–*Leishmania* associations was assessed using 40 midguts; thus, 160 in vitro interactions were performed.

**Fig. 1:**
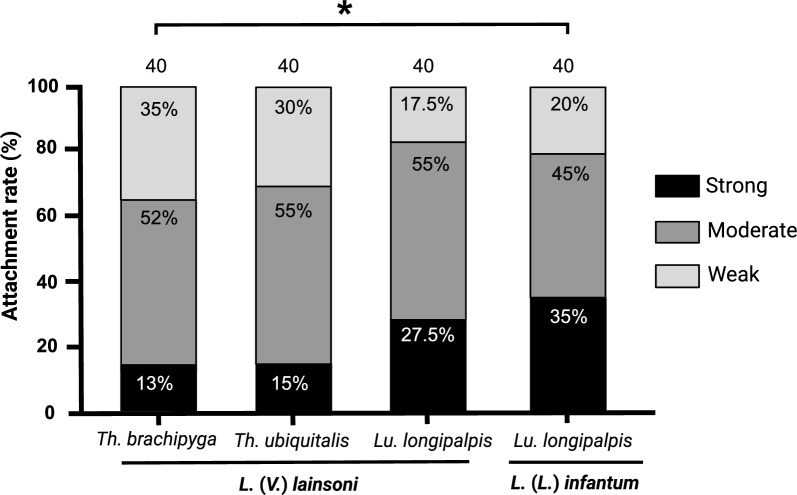
In vitro interactions between *Trichophoromyia brachipyga*–*Leishmania* (*Viannia*) *lainsoni*, *Th. ubiquitalis*–*L.* (*V.*) *lainsoni*, and *Lutzomyia longipalpis*–*L.* (*V.*) *lainsoni*. The susceptible association *Lu. longipalpis*–*L*. (*L*.) *infantum* was used as a control. The attachment profiles were classified into three categories: weak (less than 100 parasites per gut), moderate (100–1000 parasites per gut), and heavy (more than 1000 parasites per gut); values inside the bars indicate percentages of each category; values on the bars indicate the number of guts assessed. ^*^P < 0.05

Attachment profiles of sand flies with *L.* (*V.*) *lainsoni* and *Lu. longipalpis* with* L*. (*L*.) *infantum* with the respective intensity percentages (weak, moderate, and heavy) are presented in Fig. 1. There was no difference between the attachment profiles in the following comparisons: *Th. ubiquitalis*–*L.* (*V*.) *lainsoni* versus *Lu. longipalpis*–*L.* (*V*.) *lainsoni*–*L.* (*L*.) *infantum* (Table 1). Despite the limitations of in vitro infection assays, the similar adhesion profile to that of *Lu. longipalpis*, a species known to be permissive [19], provides initial experimental evidence for investigating the potential role of *Th. ubiquitalis* in the transmission cycle of cutaneous leishmaniasis caused by *L.* (*V.*) *lainsoni*, widely described in the literature [11]. However, additional in vivo susceptibility assays with different *Leishmania* spp. are essential.

**Table 1:** Summary statistics for the comparison of in vitro binding in different parasite–vector combinations

Sand fly species	*Leishmania* spp.	N	Statistics
*Th. ubiquitalis*	*L.* (*V.*) *lainsoni*	40	G test = 2.8240; *df* = 2; *P* = 0.2437
*Lu. longipalpis*		40	
*Th. ubiquitalis*	*L.* (*V.*) *lainsoni*	40	G test = 4.4974; *df* = 2; *P* = 0.1055
*Lu. longipalpis*	*L.* (*L.*)* infantum*	40	
*Th. brachipyga*	*L.* (*V.*) *lainsoni*	40	G test = 4.7078; *df* = 2; *P* = 0.0950
*Lu. longipalpis*		40	
*Th. brachipyga*	*L.* (*V.*) *lainsoni*	40	G test = 6.3272; *df* = 2; ***P* = 0.0423**
*Lu. longipalpis*	*L.* (*L*.)* infantum*	40	

Significant differences are highlighted in bold

Interestingly, the adhesion profile of *Th. brachipyga*–*L*. (*V*.) *lainsoni* was similar to that of *Lu. longipalpis*–*L*. (*V*.) *lainsoni* but weaker than that of the *Lu. longipalpis*–*L*. (*L*.) *infantum* control (Fig. 1; Table 1), a classical parasite–vector binomial association [19, 22]. Considering that the success of an infection depends, in the first instance, on the establishment of the parasite’s attachment to the sand fly midgut [16], attachment differences herein observed may converge with the hypothesis that the natural infection of *Th. brachipyga* appears to be more occasional or still emerging, without ruling out a possible role as an alternative or secondary vector [11, 14].

There was no difference between the adhesion profiles of *Lu. longipalpis* with *L.* (*V*.) *lainsoni* and *L*. (*L*.) *infantum*. However, when Sánchez-Uzcátegui et al. [30] compared the susceptibility of *Lu. longipalpis* with these *Leishmania* spp. on the eighth day post-blood meal (pbm), they observed greater parasitosis with *L*. (*L*.) *infantum*. This suggests that, although the initial adhesion profiles are proportionally similar, the maintenance during late-stage infections is better established when considering the natural association between sand flies and *Leishmania* spp.

Furthermore, the development of infection in the vector may also be related to the growth characteristics of the species involved in the infection [34]. Aspects of the molecular challenges imposed by the vector’s digestive environment and the adaptations developed by *Leishmania* parasites have already been reviewed [35]. Still, in this regard, the present authors are aware of limitations. As stated by Wilson et al. [28], in several vector–parasite pairs, the specificity of in vitro binding alone is insufficient to explain overall vector specificity since other significant barriers to development must exist in certain refractory *Leishmania*–sand fly vector combinations.

### Glycoconjugate detection

**Fig. 2:**
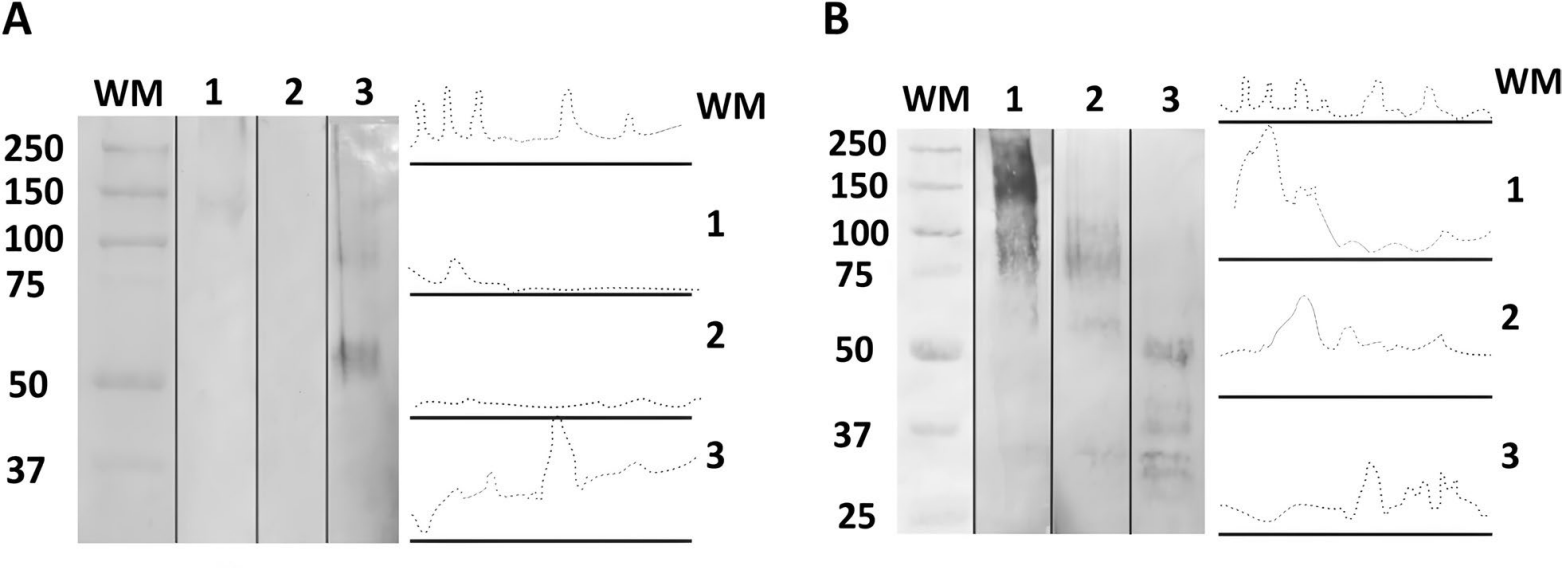
Western blot and densitometry analyses of peroxidase-conjugated lectin reaction profiles for the detection of sand fly gut glycoconjugates. **A** Peanut agglutinin (PNA), indicating galactose/GalNAc (Gal/GalNAc); **B** *Helix pomatia* agglutinin (HPA), indicating *N*-acetyl-d-galactosamine (GalNac). 1 indicates *Trichophoromyia ubiquitalis*; 2 indicates *Th. brachipyga*; 3 indicates *Lutzomyia longipalpis*. WM, molecular weight marker (values in kilodaltons)

To evaluate the presence of possible ligands for *Leishmania* in the sandfly species tested, each carbohydrate detection (GlcNAc, Gal/GalNAc, and GalNAc) was performed in triplicate with seven pooled midgut samples of three sand fly species; thus, 189 midgut samples were used. Regarding intestinal glycoconjugates, the WGA lectin showed no reactivity in any of the species tested, while PNA lectin was reactive only in *Th. ubiquitalis* and *Lu. longipalpis*. In *Th. ubiquitalis*, the reactivity was weaker, presenting bands of ~150–250 kDa, whereas the *Lu. longipalpis* presented the highest labeling reactivity, with bands ranging from ~50 to 250 kDa (Fig. 2). The lack of PNA reactivities in *Th. brachipyga* and WGA in all species suggest that Gal/GalNAc and GlcNAc residues are not terminally exposed in the midgut glycoconjugates of the sand fly species tested, making them inaccessible to lectins. The absence of terminal GlcNAc residues apparently did not affect promastigote adhesion, as the parasite interactions with the permissive vector *Lu. longipalpis* predominantly exhibited strong and moderate adhesion, and only comparison with *Th. brachipyga* showed significant differences. Similarly, the absence of terminal Gal/GalNAc residues in *Th. brachipyga* alone does not appear to be sufficient to reduce adhesion, as observed in comparison with other vector–parasite systems that possess this terminal carbohydrate. Therefore, it is unlikely that these sugar residues act as binding sites for the tested *Leishmania* species, even if ligands for these carbohydrates are possibly present in *Leishmania* spp. [36].

*N*-Acetyl-d-galactosamine (GalNAc) is particularly important, as it was identified as a component of glycoconjugates present exclusively in the intestines of permissive sand flies and later demonstrated as part of the midgut mucins capable of binding to *Leishmania,* possibly by a lectin-like molecule present on the surface of the promastigote [17, 37]. Our data demonstrated HPA lectin reactivity in all tested sand fly species. *Th. ubiquitalis* presented greater reactivity, with bands ranging from ~70 to 250 kDa. In *Th. brachipyga,* the reactivity was slightly lower and presented bands ranging from ~60 to 110 kDa (Fig. 2). In *Lu. longipalpis*, the intensity was lower than that of *Th. ubiquitalis* but close to *Th. brachipyga* with bands of ~30–50 kDa. The results, visually observed in the run, were confirmed by densitometry, suggesting preliminary evidence of possible permissiveness for *Th. ubiquitalis* and *Th. brachipyga*. However, the limitations of these findings should be overcome with supporting in vivo experimental data and further characterization of the *Leishmania* molecule possibly mediating this binomial interaction.

## Conclusions

The attachment profiles and glycoconjugates presented herein provide a better understanding of the interactions between the sand fly and medically important *Leishmania* spp. in the Amazon region. Accumulating, sometimes controversial data should be carefully considered when unraveling these complex and specific relationships.

## Data Availability

No datasets were generated or analyzed during the current study.
